# Digital morphine: why AI scribes are symptomatic relief for a broken system

**DOI:** 10.1136/bmjdh-2026-000030

**Published:** 2026-03-09

**Authors:** Bradley Segal, Luke Allen, Joshua Fieggen, David Clifton, Lei Clifton

**Affiliations:** 1 Department of Engineering Science, University of Oxford, Oxford, England, UK; 2 Primary Care, University of Oxford, Oxford, UK

**Keywords:** Artificial intelligence, Electronic Health Records, Primary Health Care, Speech Recognition Software

Artificial intelligence (AI) ambient scribes are one of the most rapidly adopted technologies in healthcare today. In a sector defined by its resistance to change, their meteoric rise tells a powerful story.^1^ For clinicians on the front line, this is a bottom-up revolution driven by a search for relief.^2^ Scribes are being embraced not to see more patients, but to survive the day. For many doctors, they promise a tangible reprieve from the profound cognitive load of documentation, a chance to reduce the after-hours administration that erodes personal lives, and, crucially, an opportunity for a doctor to finally look at their patient instead of a screen.

But a different narrative is playing out in many boardrooms and strategy documents. Here, scribes are conceived as a tool for addressing the UK’s intractable access crisis, with the aim of increasing patient throughput.^3^ This framing misdiagnoses the problem. AI scribes are an effective symptomatic relief for the administrative burden crushing clinicians. To mistake them for a cure risks entrenching the very dysfunctions they are treating. The current evidence is clear: scribes can modestly reduce documentation time but do not translate into increased patient throughput.^4 5^ This is notable given that most studies originate from the USA, which has the heaviest electronic health record burden^6^ and would be most likely to demonstrate throughput gains.

For the UK primary care sector, there is good reason to believe the same will hold true. The bottleneck in care is not typing speed; it is staggering complexity managed under extreme time pressure. Within a standard 10-to-15-minute primary care consultation slot, a visit brief by international standards,^7^ General practitioners (GPs) are routinely managing two or three distinct problems per visit. Nearly half the consultations in the UK meet criteria for being ‘complex’.^8^ The few minutes an AI scribe saves are not creating a new appointment in a packed schedule; they are preventing the current one spilling into a clinician’s evening. This reality, coupled with the technology’s risks of generating bloated or inaccurate notes and enforcing review and revision, confirms that scribes are not a panacea.^9^ They are an imperfect tool providing critical support for a system stretched too thin.

The key question, therefore, is not how scribes will solve access but how to leverage the technology’s momentum to drive systemic change. To hope the solution is in accelerating the same broken model of high-volume, low-continuity care would be a profound failure of imagination. The challenge is identifying ways of reinventing processes with technology that ask a more fundamental question: why is this patient in front of a GP right now, and is this the best mechanism for delivering care?

Answering this question demands a three-part strategy: reclaiming value within the consultation, automating the invisible workload beyond it and ultimately reshaping when and why patients need to be seen at all.

First, clinicians can choose to reinvest the newfound attention directly into the clinical encounter. The capacity released by automating administrative work should enable a more flexible, needs-based system, creating the option for longer consultations for the growing number of patients with complex multimorbidity. This is the time required for the shared decision-making and relationship-building that forms the bedrock of safe, person-centred care.

Second, we must look beyond the scribe and use AI to augment the administrative ecosystem of which the clinical note is just one, highly visible part. The real prize for efficiency lies in tackling the vast, ‘invisible’ workload that crushes practices: the relentless stream of hospital letters to be processed, repeat prescriptions to be managed and test results to be actioned. Intelligent systems that can automatically parse, summarise and action this torrent of information would release far more capacity than scribing alone. Future scribes must evolve to tackle this space that has been sorely under-investigated.^10^

Third, AI can reshape not just what happens within the consultation but also whether and when one is needed at all. Intelligent systems could monitor patient populations for preventive opportunities, pre-emptively order routine screenings or use conversational interfaces to gather history before a visit. Parallel access routes can be created for care that does not require direct clinical oversight. If needs are identified earlier and preparation is more thorough, fewer consultations become urgent firefighting exercises. The clinical encounter transforms from a data-gathering bottleneck into a focused event where GP time is spent on diagnosis and shared management.

AI scribes alone will not fix healthcare. They are welcome relief for a workforce under siege, but we need to go further. If we treat the breathing space they offer not as a solution, but as the first step in a course of treatment, they can help us begin to restore a more sustainable, effective and accessible person-centred system of care.

## Data Availability

No data are available.
